# Microencapsulation of *Cymbopogon citratus* D.C. Stapf Essential Oil with Spray Drying: Development, Characterization, and Antioxidant and Antibacterial Activities

**DOI:** 10.3390/foods11081111

**Published:** 2022-04-13

**Authors:** Denise Dantas de Oliveira Alencar, Evandro Leite de Souza, Erika Thayse da Cruz Almeida, André Leandro da Silva, Hugo Miguel Lisboa Oliveira, Mônica Tejo Cavalcanti

**Affiliations:** 1 Post-Graduation Program in Agroindustrial Systems, Center for Agro-Food Science and Technology, Federal University of Campina Grande, Pombal 58840-000, PB, Brazil; denisedantas.d@gmail.com (D.D.d.O.A.); monicatejoufcg@gmail.com (M.T.C.); 2 Laboratory of Food Microbiology, Department of Nutrition, Health Sciences Center, Federal University of Paraíba, João Pessoa 58051-900, PB, Brazil; erika_yse@hotmail.com; 3 Center for Health and Rural Technology, Federal University of Campina Grande, Patos 58708-110, PB, Brazil; andre.leandro@ufcg.edu.br; 4 Post-Graduation Program in Food Engineering, Academic Unity of Food Engineering, Federal University of Campina Grande, Campina Grande 58429-900, PB, Brazil; hugom.lisboa80@gmail.com; 5 National Institute of Semi-Arid, Ministry of Science, Technology and Innovations, Campina Grande 58434-700, PB, Brazil

**Keywords:** encapsulation, lemongrass, characterization, biological activities

## Abstract

This study aimed to microencapsulate *Cymbopogon citratus* essential oil (CCEO) with spray drying using maltodextrin and gelatin. The effects of the operational conditions (inlet temperature (130–160 °C), CCEO concentration (5–15%), maltodextrin concentration (10–20%)) on the physicochemical stability and antioxidant and antibacterial activities of the CCEO microcapsules were determined. The CCEO microencapsulation process had yield and encapsulation efficiency values varying from 31.02 to 77.53% and 15.86–61.95%, respectively. CCEO microcapsules had antibacterial effects against *Staphylococcus aureus* and *Escherichia coli* with minimum inhibitory concentration varying from 10 to 20%, and total phenolic contents and antioxidant activities varying from 1632 to 4171.08 μg TE/g and 28.55–45.12 µg/g, respectively. CCEO microcapsules had average diameters varying from 5.10 to 10.11 µm, with spherical external structures without cracks and apparent pores. The best desirable process conditions for CCEO microencapsulation were process inlet temperature of 148 °C, maltodextrin concentration of 15%, and CCEO concentration of 10%. The results showed that CCEO microcapsules with increased stability and low degradation of active components can be prepared by spray drying using maltodextrin and gelatin with the production of microcapsules, which could be exploited as potential food preservatives.

## 1. Introduction

Essential oils have been widely studied due to their diverse biological activities and well-reported potential for application as green preservatives and flavoring agents in foods. The well-established antimicrobial, antioxidant, anti-inflammatory, and analgesic properties of various essential oils [1] have resulted in increased demand of consumers for added-value and “green” products containing these substances. The essential oil extracted from the plant species *Cymbopogon citratus* D.C. Stapf. (lemongrass) has shown antimicrobial and antioxidant activities in vitro and in vivo [2,3], as well as when applied directly into different foods or comprising edible coatings designed for food application [4,5].

The essential oil from *C. citratus* accounts for more than 75% of the plant weight and is cited as generally considered as safe (GRAS) at the doses typically used in foods [5]. However, the bioactive components found in *C. citratus* essential oil are highly volatile, poorly soluble in aqueous media, sensitive to heat and oxidation in the presence of light, air, and moisture, besides being sensitive to degradation under the influence of environmental conditions [6]. These characteristics could represent important limitations to the effective use of CCEO in strategies to preserve foods.

Microencapsulation has been considered a strategy to provide increased stability, better dispersion properties, maintenance of biological activities, and protection from possible detrimental effects caused by external agents in essential oils [7]. Microencapsulation is a technology that enables the coating of solid, liquid, or gaseous materials into microcapsules where the active substances (core materials) are coated with secondary substances (wall material or encapsulating agents). Among the microencapsulation methods, spray drying has been considered one of the most common encapsulation techniques [8]. The expansion of the use of the spray drying microencapsulation process has been related to its low operational cost, large-scale continuous production, high encapsulation efficiency, and prolonged shelf life, protection against degradation, and volatilization of the constituent materials forming the obtained powders [9].

Few studies have focused on the production of microcapsules of *C. citratus* essential oil using spray drying. Some studies have evaluated the use of acacia gum, maltodextrin from corn and cassava, modified starch, and/or cyclodextrins as wall materials alone or in combination, as well as their influence on the quality of the encapsulated material [10,11]. Maltodextrin as a wall material offers advantages, such as relatively low cost, no aroma or taste, low viscosity, and good protection against oxidation. However, the biggest problem of this wall material is its low emulsifying capacity; therefore, it should be combined with a protein, such as gelatin [12,13]. However, little has been investigated regarding the effects of the variables of the microencapsulation process by spray drying on the efficiency of the process and characteristics related to the stability and bioactive properties of the obtained powder. The determination of the best operational conditions of the CCEO microencapsulation process by spray drying, which is a technique commonly used by the food industry, could contribute to the higher stability of this essential oil and help to promote a more efficient application as a green food preservative.

This study aimed to perform microencapsulation of CCEO by spray drying using maltodextrin and gelatin as wall materials, as well as to investigate the effects of the operational conditions of the process (drying air temperature at the inlet, essential oil concentration, and maltodextrin concentration) on the physicochemical stability and antioxidant and antibacterial properties of the microencapsulated essential oil to be used as a food additive. The best desirable process conditions for CCEO microencapsulation were also determined.

## 2. Materials and Methods

### 2.1. Materials

The CCEO extracted from the leaves of the plant species (*C. citratus* D.C. Stapf.) by steam distillation was used as the core material. The CCEO was obtained from Quinarí Ind. Com. Ltd.a. (Ponta Grossa, PR, Brazil). Maltodextrin DE20 (Tate & Lyle, Indianópolis, PR, Brazil) and gelatin (Bretzke, Jaraguá do Sul, SC, Brazil) were used as encapsulating materials. Tween 80 (Sigma Aldrich, Saint Louis, MA, USA) was used as an emulsifying agent. The identification of the CCEO constituents was performed by gas chromatography coupled to mass spectrometry technique, according to previously described analytical procedures [14]. Geranial (21.85%) was identified as the most prevalent constituent in the tested CCEO, followed by neral (17.33%), geranyl acetate (4.91%), and linalool (2.08%).

### 2.2. Emulsion Preparation

The methodology described by da Silva Martins et al. [13] was used for the emulsion preparation. Initially, the wall materials were weighed, and maltodextrin and gelatin (rate 9:1, *w*/*w*) [13] were used to compose the encapsulating matrix. The mixture was dissolved in distilled water (25 ± 1 °C) and kept under stirring until complete dissolution, followed by the addition of Tween 80 as a surfactant in an amount corresponding to 5% (*v*/*w*) of the encapsulants mass (wall materials). The CCEO was added directly into the mixture and stirred for 5 min with a Vortex. The concentrations of maltodextrin and CCEO varied according to the experimental design used, but the maltodextrin:gelatin ratio was fixed at 9:1 (*w*/*w*). Gelatin and Tween 80 were included due to the poor emulsification properties of maltodextrin [12,13].

### 2.3. Spray Drying Process

The drying was performed using a co-current spray dryer (Labmaq, model SD-10, Ribeirão Preto, SP, Brazil) with a 1.5 mm diameter atomizer nozzle and cyclone for powder collection on an open loop. Atmospheric air with 0.014 g H_2_O/g air was withdrawn without any treatment and blown at a constant drying gas flow rate of 500 kg/h. The inlet temperature was adjusted according to the design of experiments (Table 1). The spray dryer was fed with a peristaltic pump (PD 5201, Heidolph Instruments, Schwabach, Germany) with feed flowrate of 4 kg/h. The atomization was performed with a two-fluid nozzle where atmospheric air was used at a flowrate of 4 kg/h.

### 2.4. Experimental Design

A 3^3−1^ factorial design was used, totaling nine trials. The experimental design was carried out to improve the encapsulation process and evaluate the effects of the independent variables within the following variations: inlet temperature (130–160 °C), CCEO concentration (5–15%, *v*/*w*), and maltodextrin concentration (10–20%, *w*/*w*). The levels and values of the independent variables used in the experimental design and the generated matrix are shown in Table 1 and Table 2, respectively. The variables were chosen after previous work [12].

### 2.5. Characterization of the Microcapsules

#### 2.5.1. Microencapsulation Performance

The microencapsulation performance was based on the masses of the encapsulants, CCEO, and surfactant used to prepare the emulsions and the final mass after the spray drying, and calculated with the following equation [15]:

(1) $$\mathrm{MP}=\frac{M_{\mathrm{final}}}{M_{\mathrm{initial}}}\times 100$$

where MP: microencapsulation performance; M_final_: mass of the microencapsulated product after spray drying; and M_initial_: dry mass of the encapsulants, essential oil, and surfactant.

#### 2.5.2. Microencapsulation Efficiency

The microencapsulation efficiency, which indicates the ability of the essential oil to be retained by the encapsulating matrix, was determined based on the content of the inserted CCEO and the content retained after the process. The percentage of CCEO retained in the microcapsules was established according to a previously described methodology [16,17]. The encapsulation efficiency was calculated with the following equation:

(2) $$\mathrm{ME}=\frac{{\mathrm{EO}}_{\mathrm{real}}}{{\mathrm{EO}}_{\mathrm{theoretical}}}\times 100$$

where ME: microencapsulation efficiency; EO real: essential oil content retained; and EO theoretical: essential oil content inserted.

#### 2.5.3. Moisture

Approximately 3 g of the microcapsule samples were weighed into an aluminum crucible previously weighed, tared, and cooled in a desiccator to avoid moisture absorption, and, consequently, inducing errors in the results. The samples remained in an oven for 24 h at 105 °C, placed in a desiccator, and finally weighed on an analytical scale. Moisture calculations were obtained with the following equation [18]:

(3) $$M\;\left(\%\right)=\frac{\left(M_{c}-M_{i}\right)-M_{f}}{\left(M_{c}-M_{i}\right)}\times 100$$

where M_c_: mass of the crucible without a sample (g); M_i_: mass of the crucible with a sample, initial (g); and M_f_: mass of the crucible with a sample, final (g).

#### 2.5.4. Water Activity

The water activity was determined using a water activity analyzer (Aqua Lab, Model 4TE, Meter Group Inc., Riverside, CA, USA) under constant temperature (25 °C) [19].

#### 2.5.5. Color

The instrumental color parameters were set using a MiniScan XE Plus colorimeter (Hunter Associates Laboratory Inc., Reston, VA, USA) in a function suitable for powder (Color Plot D65/10°), following the procedures indicated by the manufacturer for setting the values of L* (lightness), a* (red/green coordinate, where +a indicates red and -a indicates green), and b* (yellow/blue coordinate, where +b indicates yellow and -b indicates blue).

#### 2.5.6. Ultrastructural Aspects and Particle Distribution of the Microcapsules

The ultrastructural aspects of the microcapsules were evaluated with scanning electron microscopy (SEM) using Tescan Vegan 3 equipment (Tescan Orsay, Kohoutovice, Czech Republic) operating at 5 kV, high vacuum, and magnification ranging from 50× to 2000×. No coating of the microcapsules was used for the analysis. To set the average particle size distribution, SEM images of the CCEO microcapsules were analyzed using the ImageJ software (National Institute of Health, Bethesda, MD, USA). The parameter D_n_50, which represents the average size of the microcapsules and indicates that 50% of the particle size is smaller than the value presented, was calculated using a previously described methodology [10].

### 2.6. Determination of the Total Phenolic Content and Antioxidant Activity of Microcapsules

The total phenolic content was determined with the Folin–Ciocalteu method [20] using a UV-visible spectrophotometer (Model SP-220, Biospectro, Curitiba, PR, Brazil) with absorbance measured at 765 nm. A standard curve was drawn with a gallic acid solution (0.1 g/L), and the results were expressed as gallic acid equivalents (µg of gallic acid/100 g of extract).

The antioxidant activity was evaluated based on the measurement of the DPPH (2,2-diphenyl-1-picrylhydrazyl) free radical scavenging activity [21]. The calibration curve was completed with solutions of DPPH (75 µM), Trolox (500 µM), and distilled water, with final concentrations of 17.5, 35, 70, 105, 140, and 175 mg/mL. The extract was prepared with 2 g of the microcapsules (0.2 g of CCEO) diluted in 10 mL of methanol, and the control was prepared with a solution of 3150 µL of DPPH, 200 µL of distilled water, and 150 µL of the extract. The reduction of the DPPH radical was measured by reading the absorbance at 515 nm, at time zero and after 30 min of reaction, using the equation y = 2.8821x + 7.732. The results were expressed as µg of Trolox equivalent per g of extract sample (µg TE/g).

### 2.7. Evaluation of the Antibacterial Activity of Microcapsules

The antimicrobial activity of the CCEO microcapsules and pure CCEO was evaluated by determining the minimum inhibitory concentration (MIC) against *E. coli* (ATCC 11775) and *S. aureus* (ATCC 13565) using microdilution in broth. Aliquots of the CCEO stock solutions (100 μL) and CCEO microcapsules (15 µg/mL) were prepared according to a previously described procedure [22], dispensed and homogenized into the wells (first row) of a 96-well microplate containing 100 μL of brain heart infusion broth (BHI, HiMedia, Mumbai, India). Subsequently, aliquots of 100 μL contained in the first-row wells were transferred to the following wells (second row) using geometric dilutions of ratio two. Aliquots of 100 μL of the tested bacterium inoculum suspension were added to each well (final viable cell counts of approximately 2 × 10^8^ CFU/mL) to obtain eight different final concentrations for the microencapsulated and pure CCEO, to cite: 7.5, 3.75, 1.875, 0.937, 0.468, 0.234, 0.117, and 0.058 μg/mL. In each microplate, a positive control (inoculated BHI) and a sterility control (uninoculated BHI) were used for each strain tested. The microplate was incubated at 30 °C for 24 h. Resazurin, an oxy-reduction indicator, was used to perform the reading of the results, in which 40 µL of resazurin solution (100 µg/mL) was added to the wells. The appearance of the blue and pink colors was considered indicative of the absence and presence of microbial growth, respectively. The MIC was considered as the lowest microencapsulated and pure CCEO concentration capable of inhibiting the visible growth of the tested bacterial strain at the end of the incubation period.

### 2.8. Determination of the Best Desirable Process Conditions for the CCEO Microencapsulation Process

To perform the combination among the levels of the operational parameters (inlet temperature, maltodextrin concentration, and CCEO concentration) and find the best conditions to produce the most desirable responses for the encapsulation efficiency, antioxidant activity, and total phenolic content, the global desirability function was used. This function allows normalizing the values obtained from the individual desirability (d_i_) values and permits obtaining the overall desirability value (D) for each trial, calculating the geometric mean of the values. The desirability values are presented on a scale from 0 to 1, where values close to 1 are considered desirable [7].

### 2.9. Analysis of the Experimental Data

The results of all characterization analyses were modeled based on the values of the independent variables according to the experimental design. A second-degree polynomial equation was used, where the experimental results of each dependent variable were adjusted by non-linear regression. The obtained models were evaluated using ANOVA at 5% probability (*p* < 0.05). All calculations were completed using the STATISTICA 12.0 software (Tibco Software Inc., Palo Alto, CA, USA). All the experiments were completed in triplicate on three separate occasions.

## 3. Results and Discussion

### 3.1. Process Parameters and Chemical Characteristics of CCEO Microcapsules

To assess the CCEO microencapsulation process by spray drying, a 3^3−1^ experimental design was run with inlet temperature, maltodextrin concentration, and CCEO concentration as independent variables. The parameters yield, water activity, moisture, average particle size distribution, color, encapsulation efficiency, total phenolic content, and antioxidant activity were used as dependent variables (responses) (Table 3 and Table 4).

The yield is a parameter related to the cost–benefit of production and it is an important aspect for monitoring the spray drying process. The process applied for CCEO microencapsulation resulted in yield values between 31.02 and 77.53%. The yield of drying operation considered successful should be close to 50% [23], which indicates that experiments E.6 (62.58%) and E.7 (77.53%) resulted in the best yields among the CCEO microencapsulation process conditions evaluated.

None of the independent variables analyzed significantly (*p* > 0.05) affected the yield of the CCEO encapsulation process. However, although it was not possible to identify significant trends, the lower yields of the process resulted from the application of the lower inlet temperature in the drying process, which may be related to the lower efficiency of heat and mass transfer. It can be suggested that the temperature employed in the drying process was not effective enough to cause the evaporation of the water before the material met the drying chamber walls, making it possible for part of the material to adhere to the chamber causing lower performance. A similar effect was reported in a previous study with Buriti oil microencapsulation in chickpea protein/pectin matrix by spray drying [8].

The encapsulation efficiency (EE) is one of the most important parameters for the encapsulation of essential oils because it refers to the percentage of the substance that has become encapsulated as part of the core of the obtained material. The EE value indicates how effective the drying conditions were since ultimately the objective of the spray drying process is to encapsulate the oil. Higher values of EE reveal that the drying process conditions minimize volatilization losses. The EE values for the different CCEO encapsulation formulations ranged from 15.86 to 61.95%, which were close to those found in a study on walnut oil encapsulation by spray drying (22.52–56.19%) [24].

The EE was highly influenced (*p* < 0.05) by maltodextrin concentration, as well as by the interaction between CCEO concentration and maltodextrin concentration. A proportional relationship was found between maltodextrin concentration and EE value since the use of a higher concentration of the encapsulating agent increased the EE value. A similar result was found in the process of microencapsulation of *Lavandula officinalis* (lavender) essential oil by spray drying. Higher concentrations of *L. officinalis* essential oil resulted in lower EE, which was related to the reduction in the capacity of the encapsulating materials to coat the essential oil droplets as it increased the charge in the emulsion used in the process [25].

It can be suggested that the use of higher concentrations of maltodextrin resulted in higher CCEO retention capacity in the microcapsules. Maltodextrin has a higher molecular weight than CCEO, causing a lower diffusion velocity when drying the emulsion droplet. It results in a greater possibility of maltodextrin forming a crust in more superficial regions than the CCEO in the emulsion droplet, resulting in particles with lower superficial essential oil content and, consequently, in lower EE values for the drying process. The decrease in maltodextrin concentration in the emulsion formulation may have caused less crust to be formed in the droplet surface and, consequently, less CCEO retention in the microsphere and its loss due to volatilization during the spray drying [26].

The values obtained for the moisture content of the microcapsules containing CCEO ranged from 1.55% to 10.76%. These moisture values were similar to those reported in *Nigella sativa* (black cumin) essential oil microcapsules obtained by spray drying [26]. The moisture content is an important influential parameter for the stability of encapsulated products during storage and is directly related to the fluidity of the powder and drying efficiency [26,27]. Among the effects monitored, moisture content had a significant influence on the amount of CCEO used in the microcapsule formulation (*p* < 0.05). Probably, the high CCEO content may have allowed obtaining microcapsules with a greater amount of surface essential oil, causing the formation of a barrier for water diffusion inside the particle and reducing evaporation, resulting in more humid particles. It is also possible to identify a clear trend between the samples with higher EE and moisture content. Therefore, the use of higher CCEO concentration in the formulation combined with lower maltodextrin concentrations and lower temperatures resulted in CCEO microcapsules with higher moisture contents.

The temperature of entry into the drying process also influenced the moisture content of the microcapsules. Thus, the use of higher drying temperatures resulted in obtaining CCEO microcapsules with lower moisture values. The use of higher inlet temperatures may have resulted in a higher heat transfer rate to the particles; thus making the evaporation rate higher, which resulted in obtaining microcapsules with lower moisture content. This behavior was also observed for the microencapsulation of *Citrus* × *aurantifolia* (lime) essential oil by spray drying, where the lowest moisture contents were obtained by using a higher inlet temperature (180 °C) [28].

The water activity (a_w_) values obtained for the microcapsules were low and ranged from 0.06 to 0.22, which were similar to those found in previous investigations with microencapsulation of different essential oils [9]. The a_w_ values are linked to the chemical and microbiological stability of microencapsulated products. In regions with a_w_ values below 0.3, the water within the product is strongly bound, which inhibits microbial growth and reduces the relative rate of oxidative reactions, aiding in the safety and stability of the product [29]. Therefore, the low a_w_ found in all microencapsulated CCEO samples indicate their stability for storage.

The CCEO concentration, as well as the interaction between temperature and CCEO concentration, significantly influenced (*p* < 0.05) the a_w_ values in the microencapsulated samples. The increased concentration of CCEO caused an increase in a_w_ values, which was behavior like that found for moisture. Higher CCEO concentrations possibly resulted in a higher amount of surface oil in the emulsion used for drying, hindering water evaporation, and generating wetter particles [27].

Color, as a parameter directly linked to visual attractiveness, is a relevant aspect for the acceptance of encapsulated materials. All CCEO microencapsulated samples showed light color, with L* values close to 100. The b* coordinate represents the blue to yellow scale, with negative values for blue and yellow for positive ones. All samples showed positive values, indicating the predominance of yellow intensity. The different conditions employed in the microencapsulation process had no significant influence (*p* > 0.05) on the color parameters evaluated. According to a study on microencapsulation of powder avocado drink by spray drying [12], the color stability of the sample indicates that the inlet temperatures used in the process did not significantly influence the samples evaluated and, consequently, did not cause darkening or caramelization due to thermal exposure, being pointed out as a very positive point.

### 3.2. Contents of Total Phenolic Compounds and Antioxidant Activity of CCEO Microcapsules

The quantification of total phenolic content in CCEO microcapsules showed values between 1632 and 4171.08 μg TE/g. CCEO concentration and interaction of inlet temperature and CCEO concentration showed significant influence (*p* < 0.05) on the total phenolic content of the microcapsules (Table 3 and Table 4). The increased CCEO concentration in the formulation caused an increase in the content of phenolic compounds, which could be expected since a greater amount of essential oil in the emulsion can generate samples with a higher content of bioactive compounds.

However, considering the weight balance between mass and content of phenolic compounds per gram of CCEO retained in the samples, the negative influence of the interaction of inlet temperature and CCEO concentration on the content of phenolic compounds in the microcapsules can be seen. This result indicates the occurrence of the degradation of CCEO components, especially in samples obtained in the drying processes with the application of higher temperatures. These results may be related to the increased volatilization or degradation of CCEO constituents during the drying process, indicating that the phenolic compounds were more stable at the lower drying temperature (130 °C). Another possible cause of the decrease in contents of phenolic compounds provoked by high spray drying temperatures may be the alteration in their molecular structure with reduced reactivity [13,15].

In addition, the phenolic compound contents of the powder samples, when compared with the phenolic content of the pure oil, showed a relatively low percentage of retention of these compounds in the microcapsules (7.8% to 38%), evidencing the occurrence of degradation of phenolic compounds when subjected to the drying process. The occurrence of this fact can be explained by the low retention efficiency of phenolic compounds, in which the drying conditions used during the emulsion spraying process were not effective enough, leaving the essential oil exposed to oxygen and temperature, and allowing the partial degradation of the content of phenolic compounds present in the essential oil encapsulated.

The high antioxidant activity of essential oils is mainly derived from phenolic compounds with hydroxyl groups capable of acting as free radical scavengers [30]. Considering that the high antioxidant activity is presumably associated with the total phenolic content [31], a relationship between total phenolic content and antioxidant properties of CCEO microcapsules can be established. To this end, the DPPH method has been widely used to determine the preliminary free radical scavenging potential of a compound or extract, and it is commonly adopted to assess chain breaking in the propagation phase of lipid oxidation [31].

The antioxidant potential values in the evaluated CCEO microcapsules, as determined by the DPPH method, ranged from 28.55 to 45.12 mg/100g. The inlet temperature used in the spray drying process influenced (*p* < 0.05) the antioxidant activity of the microcapsules so that higher inlet air temperatures resulted in microcapsules with lower antioxidant activity (Table 3 and Table 4). This same tendency has been reported in previous investigations with essential oil microencapsulation when reductions in the antioxidant activity were caused by the increase in the drying process temperature with the occurrence of oxidative reactions and loss of volatile compounds of the essential oil due to the heat employed in the process [27].

Considering the antioxidant activity of the CCEO before the drying process, and comparing it with the powder materials, the antioxidant potential of the microcapsules reduced considerably (62 to 87%). This reduction can be explained by the loss of bioactive compounds, because, due to the decrease in the phenolic content in the samples after the drying process, the antioxidant activity decreased, which was to be expected since the antioxidant properties of a material are related to the presence of phenolic compounds in the sample.

### 3.3. Antibacterial Activity of CCEO Microcapsules

The microencapsulated and unencapsulated CCEO showed inhibitory effects against *E. coli* and *S. aureus*, with MIC values between 0.117 and 0.234 µL/mL and 0.117 and 0.468 µL/mL, respectively (Table 5). Therefore, the different microencapsulated CCEO samples and unencapsulated CCEO tested showed antimicrobial effects against the target microorganisms, indicating that the microencapsulation process did not cause significant changes in the essential oil while maintaining its biological activity.

The MIC values found for the different formulations of microencapsulated CCEO were similar against *E. coli*. In turn, there was variation for the MIC values against *S. aureus*, where samples with higher EE percentage showed lower MIC values. This trend is consistent with the results of an early study [31] that reported the antibacterial effect of oregano essential oil encapsulated by spray drying, where the processing temperature did not affect the antimicrobial activity against *E. coli*. However, the antibacterial activity was reduced against *S. aureus* in samples encapsulated at higher processing temperatures. The authors reported that the inhibition of the growth of microorganisms is conferred by the bioactive compounds. Therefore, the different responses found are due to the thermal stability and/or diffusion capacity of these compounds, indicating the drying temperature as one of the factors that significantly influence the antimicrobial activity of microencapsulated essential oils.

The MIC values found for the different formulations of microencapsulated CCEO were similar against *E. coli*. In turn, there was variation in the MIC values against *S. aureus*, where samples with higher EE percentage showed lower MIC values, indicating stronger antimicrobial effects. These results may be related to the higher retention efficiency of the active compounds of CCEO in the samples with higher EE, which indicates a higher amount of essential oil retained in the microcapsule.

The lower MIC values were found for the different formulations of microencapsulated CCEO and pure CCEO against *S. aureus* (Gram-positive) when compared to *E. coli* (Gram-negative). This result possibly occurred due to the greater sensitivity of Gram-positive bacteria to the constituents found in CCEO. It may be related to the presence of hydrophilic liposaccharides in the outer membrane of Gram-negative bacteria, creating a barrier for the diffusion of active components in this cellular structure, hindering the action of CCEO in the target sites of the bacterial cell [32]. This is in agreement with a previous study on the inhibitory effects of microcapsules of red ginger (*Zingiber officinale* var. *Rubrum*) essential oil, which reported that Gram-negative bacteria (*E. coli*) are more resistant to the essential oil than Gram-positive bacteria (*S. aureus*) [33].

### 3.4. Microstructure of CCEO Microcapsules

**Figure 1:**
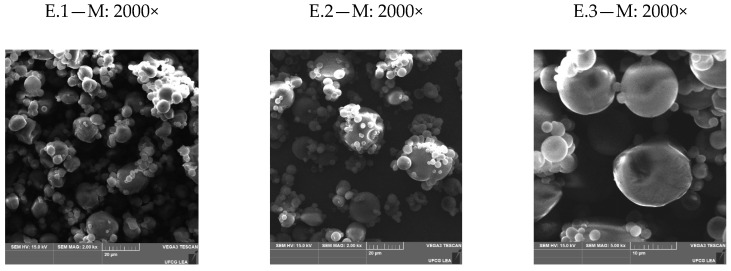

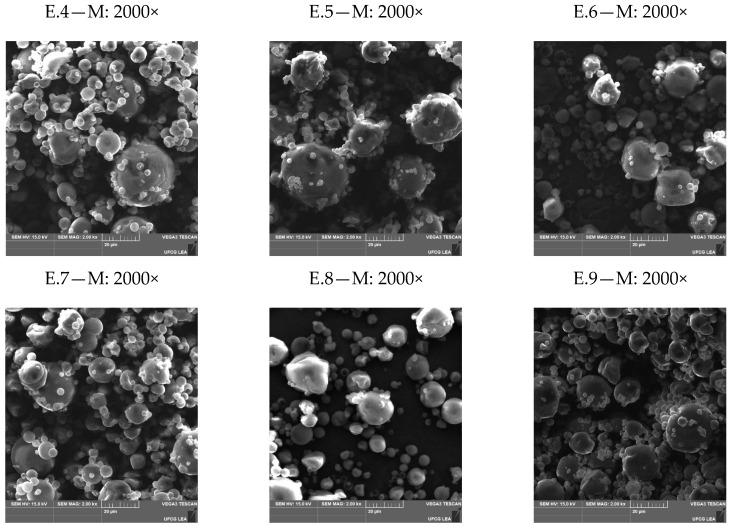
Microstructure (morphology) of the *C. citratus* essential oil (CCEO) microcapsules prepared with spray drying using maltodextrin and gelatin. T: inlet temperature; MC: maltodextrin concentration (%, *w*/*w*); CCEOC: *C. citratus* essential oil concentration (%, *v*/*w*); M: magnification. E.1 = T: 130 °C; MC: 10%; CCEO: 5%; E.2 = T: 130 °C; MC: 15%.; CCEO: 15%; E.3 = T: 145 °C; MC: 10%; CCEOC: 15%; E.4 = T: 145 °C; MC: 15%; CCEO: 10%; E.5 = T: 145 °C; MC: 20%; CCEO: 5%; E.6 = T: 160 °C; MC: 10%; CCEO: 10%; E.7 = T: 160 °C; MC: 15%; CCEO: 5%; E.8 = T: 160 °C; MC: 20%; CCEO: 10%; E.9 = T: 160 °C; MC: 20%; CCEO: 15%. The maltodextrin:gelatin ratio was fixed at 9:1.

The micrograph images of the CCEO microcapsules are shown in Figure 1. The obtained CCEO microcapsules showed similar morphology despite the application of different drying conditions. The microcapsules had a spherical external surface, and were slightly wrinkled, without cracks and apparent pores, supporting the high encapsulation efficiency of the spray drying process. Microcapsules without cracks and pores exhibit lower gas permeability and protection against oxidation reactions [15]. The combination of the encapsulants maltodextrin and gelatin offered good protection for the encapsulated CCEO. These results may be related to the mechanical properties of this mixture of encapsulants that enable it to resist the internal pressure caused by moisture vaporization. The particles formed had different sizes, since the polydispersion of the particle sizes is a typical characteristic of spray drying. In addition, agglomeration of small particles was observed, which coalesced with the surface of larger particles. The agglomeration of particles may be related to the presence of free essential oil on the surface of the encapsulated material [34].

The images obtained by SEM also allow verifying the size and distribution of the particles of the encapsulated material. The parameter D_n_50 indicates that 50% of the average diameter of the powder particles obtained in the spray drying process had smaller sizes than indicated, representing an important parameter that directly influences the appearance and fluidity of the powder. The D_n_50 values obtained for the CCEO microcapsules ranged from 5.1 to 10.11 µm.

None of the process variables significantly affected (*p* > 0.05) the D_n_50 parameter. However, there was a tendency regarding the effect of the temperatures used in the process on the average particle diameter since the increase in the inlet temperature resulted in higher D_n_50 values for the particles. Higher drying air temperatures increase the evaporation rate, and the heat transfer from the heated air to the droplets makes the evaporation of the liquid more accelerated. These conditions favor the surface evaporation process of the droplet, causing rapid formation of a dry crust layer, hindering the shrinking of the particles, and, consequently, forming larger microcapsules, which may be related to the D_n_50 values obtained in this study [29].

### 3.5. Best Desirable Process Condition for CCEO Microencapsulation

**Figure 2:**
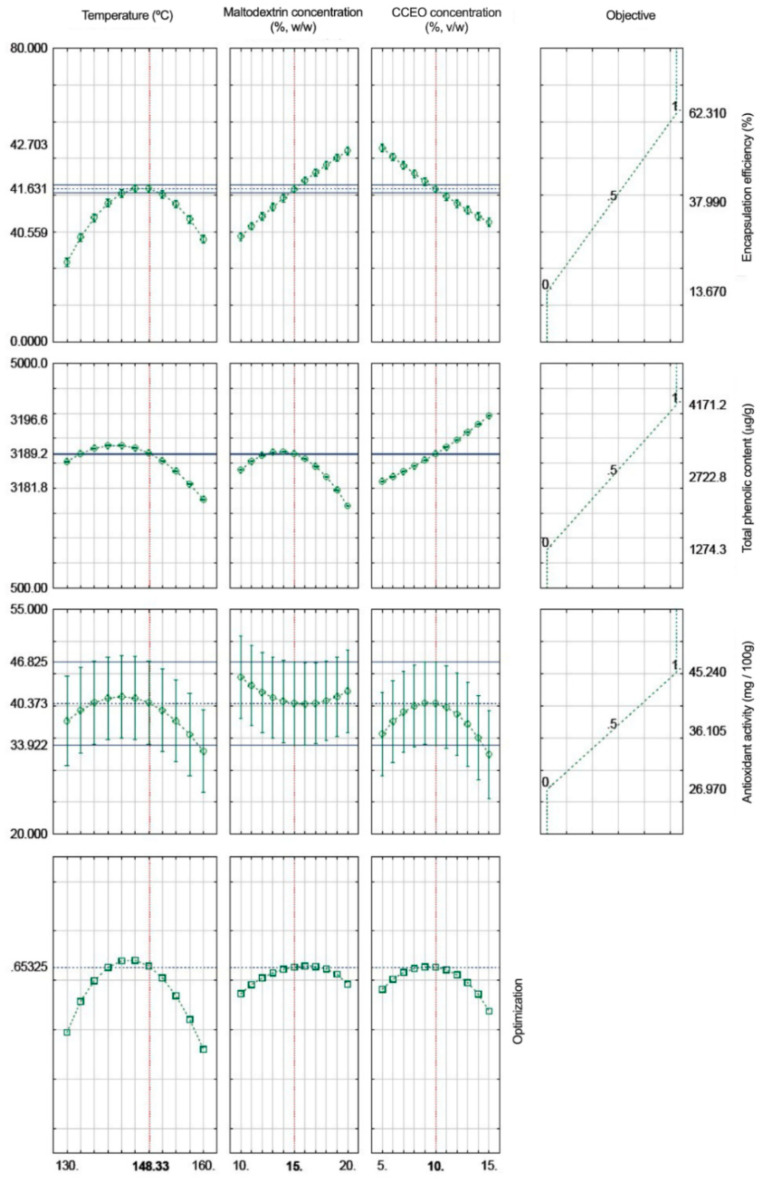
Best desirable process conditions for *C. citratus* essential oil (CCEO) microencapsulation with spray drying using maltodextrin and gelatin.

The best desirable process conditions for CCEO microencapsulation were determined by simultaneous evaluation of the best responses through desirability function analysis to achieve maximum encapsulation efficiency, total phenolic compound content, and antioxidant activity. Values were assigned from 0 to 1, where values close to 1 correspond to the maximum of each of the parameters, while values closer to 0 correspond to the least desirable conditions (Figure 2). It was found that the process inlet temperature should be 148 °C, while the maltodextrin concentration should be 15%, and the CCEO concentration should be 10% (as dependent variables) to achieve a process with 41.63% EE, 3189.189 µg/g of total phenolic content, and 40.37 mg/100 g of antioxidant activity (as independent variables). Further, validation experiments were performed to confirm the optimization results and are summarized in Table 6. Results from validation experiments were close to the values obtained when using the optimization function for each of the objectives, indicating that the optimization should be trusted.

## 4. Conclusions

The results showed that CCEO microcapsules can be prepared by emulsification using maltodextrin and gelatin as wall materials followed by spray drying. The CCEO microencapsulation process used in this study resulted in good operation yield and microencapsulation efficiency, forming spherical, poorly wrinkled microcapsules without cracks and apparent pores. Among the operating conditions of the spray drying process, the inlet temperature substantially affected the properties of the microcapsules, where higher inlet air temperature caused improvement in production yield and an increase in microcapsule size. In contrast, the use of higher inlet temperatures resulted in greater loss of phenolic compounds, which may have reduced the antioxidant activity of the CCEO microcapsules. The microencapsulated CCEO showed antimicrobial effects against *E. coli* and *S. aureus*. The best desirable process conditions for CCEO microencapsulation by spray drying included process inlet temperature of 148 °C, maltodextrin concentration of 15%, and CCEO concentration of 10%, which corresponded to maximum EE, total phenolic content, and antioxidant activity. These conditions could be considered for the large-scale production of stable and active CCEO microcapsules, but only after process intensification to increase process yield.

## Figures and Tables

**Table 1:** Levels of independent variables used in the experimental design for *C. citratus* essential oil (CCEO) microencapsulation using spray drying.

Independent variables	−1	0	1
Maltodextrin concentration (%, *w*/*w*)	10	15	20
CCEO concentration (%, *v*/*w*)	5	10	15
Inlet temperature (°C)	130	145	160

The maltodextrin:gelatin ratio was fixed at 9:1.

**Table 2:** Matrix of the experimental design for microencapsulation of *C. citratus* essential oil (CCEO) using spray drying (*n* = 3).

Experiments	Inlet Temperature (°C)	Maltodextrin Concentration	CCEO Concentration
		(%, *w*/*w*)	(%, *v*/*w*)
E.1	130 (−1)	10 (−1)	5 (−1)
E.2	130 (−1)	15 (0)	15 (1)
E.3	145 (0)	10 (−1)	15 (1)
E.4	145 (0)	15 (0)	10 (0)
E.5	145 (0)	20 (1)	5 (−1)
E.6	160 (1)	10 (−1)	10 (0)
E.7	160 (1)	15(0)	5 (−1)
E.8	160 (1)	20 (1)	10 (0)
E.9	160 (1)	20 (1)	15 (1)

The maltodextrin:gelatin ratio was fixed at 9:1.

**Table 3:** Summary of the conditions used in the spray drying process and the physicochemical characteristics and antioxidant activities of the *C. citratus* essential oil microcapsules (*n* = 3).

Experiments	T	MC	CCEOC	Yield	Moisture	a_w_	D_N_50	L*	a*	b*	EE	Content of Total Phenolic Compounds (μg/g)	Antioxidant Activity
	(°C)	(%, *w*/*v*)	(%, *v*/*w*)	(%)	(%)		(µm)				(%)		(µg TE/g)
E.1	130 ± 2	10	5	40.93	3.09 ± 0.00	0.13	5.1	72.80 ± 0.30	0.40 ± 0.01	18.00 ± 0.03	15.86 ± 0.16	1858 ± 2.40	45.12 ± 0.12
E.2	130 ± 3	15	15	31.02	10.76 ± 0.01	0.22	5.6	71.40 ± 0.02	0.80 ± 0.08	20.10 ± 0.18	18.91 ± 0.92	4171 ± 0.20	30.31 ± 1.45
E.3	145 ± 2	10	15	47.36	8.73 ± 0.00	0.14	7.02	71.10 ± 0.01	0.20 ± 0.35	18.10 ± 0.18	21.30 ± 0.24	3824 ± 2.20	39.04 ± 4.09
E.4	145 ± 3	15	10	41.4	6.30 ± 0.07	0.12	8.86	72.40 ± 0.03	0.20 ± 0.01	19.30 ± 0.10	41.78 ± 0.22	3306 ± 2.80	41.19 ± 0.24
E.5	145 ± 2	20	5	55.11	1.55 ± 0.01	0.06	8.03	71.30 ± 0.88	0.50 ± 0.04	18.40 ± 0.25	61.95 ± 0.36	1632 ± 3.60	36.63 ± 6.32
E.6	160 ± 3	10	10	62.58	2.13 ± 0.01	0.11	11.1	71.80 ± 0.80	0.10 ± 0.37	20.20 ± 0.19	13.73 ± 0.06	1898 ± 2.70	31.46 ± 0.54
E.7	160 ± 2	15	5	57.93	2.45 ± 0.06	0.1	9.77	71.80 ± 0.50	0.30 ± 0.16	19.90 ± 0.01	43.11 ± 0.58	1962 ± 6.30	28.55 ± 1.58
E.8	160 ± 3	20	10	77.53	1.73 ± 0.04	0.12	13.38	72.70 ± 0.04	0.20 ± 0.12	19.50 ± 0.01	39.71 ± 0.16	1276 ± 1.70	40.40 ± 1.45
E.9	160 ± 2	20	15	40.93	1.96 ± 0.01	0.07	10.11	70.10 ± 0.45	0.30 ± 0.26	18.80 ± 0.24	26.67 ± 0.54	1801 ± 1.70	32.05 ± 0.46

T: inlet temperature; MC: maltodextrin concentration; CCEOC: *C. citratus* essential oil concentration; D_N_50: average diameter of the particle; a_w_: water activity; EE: encapsulation efficiency. L* lightness, a* redness /greenness axis, b* blue/yellow axis. The maltodextrin:gelatin ratio was fixed at 9:1.

**Table 4:** Responses to the fit coefficients of the data by non-linear regression for the physicochemical characteristics and antioxidant activity of the *C. citratus* essential oil (CCEO) microcapsules prepared using spray drying.

Parameters	Yield (%)	Moisture (%)	a_w_	D_N_50 (µm)	a *	b *	EE (%)	Content of Total Phenolic Compounds (μg/g)	Antioxidant Activity (µg TE/g)
Intercept	98.063	−19.736	−0.069	−17.576	−0.537	−16.258	−47.938	−1747.8	261.189
T	−0.195	0.145	0.002	0.170	0.006	0.274	−0.021	30.97	−1.665 *
MC	−11.137	0.284	−0.036	−0.236	0.037	1.075	6.985 *	−536.07	−8.516
CCEO	−5.529	4.129 *	0.085 *	0.235	0.225	1.200	3.713	1598.75 *	−5.821
T*MC	0.064	−0.003	0.0001	0.002	−0.001	−0.009	−0.001	2.7	0.067
T*CCEO	0.024	−0.024	−0.001 *	−0.002	−0.002	−0.011	0.004	−10.13	0.055
MC*CCEO	0.161	−0.013	0.001	0.001	0.002	0.034	−0.411	3.17	−0.169
R^2^	91.24	97.55	92.09	84.73	83.81	90.25	91.33	88.61	72.65
Fcalc	31.91	119.75	35.65	16.65	17	27.8	31.62	23.35	7.97

T: inlet temperature; MC: maltodextrin concentration; D_N_50: average diameter of the particles; a_w_: water activity; EE: encapsulation efficiency. (*): values correspond to significance for *p* < 0.05. The maltodextrin:gelatin ratio was fixed at 9:1.

**Table 5:** The minimum inhibitory concentration (MIC, µg/mL) of the microencapsulated and pure *C. citratus* essential oil against *E. coli* and *S. aureus* (*n* = 3).

Samples (Microcapsules)				MIC (µg/mL)	
Experiments	T	MC	CCEOC	*E. coli*	*S. aureus*
	(°C)	(%, *w*/*v*)	(%, *v*/*w*)		
Ε.1	130 ± 2	10	5	0.468	0.468
Ε.2	130 ± 3	15	15	0.468	0.468
Ε.3	145 ± 2	10	15	0.468	0.468
Ε.4	145 ± 3	15	10	0.468	0.117
Ε.5	145 ± 2	20	5	0.468	0.234
Ε.6	160 ± 3	10	10	0.468	0.234
Ε.7	160 ± 2	15	5	0.468	0.117
Ε.8	160 ± 3	20	10	0.468	0.117
Ε.9	160 ± 2	20	15	0.468	0.117
CCEO	−	−	−	0.234	0.117

T: inlet temperature; MC: maltodextrin concentration; CCEOC: *C. citratus* essential oil concentration.

**Table 6:** Optimized conditions and process validation (process conditions: inlet temperature: 148 °C; maltodextrin concentration: 15%; *C. citratus* essential oil concentration: 10%).

Experiment	MIC (%, *w*/*v*)	CCEOC (%, *v*/*w*)	Encapsulation Efficiency (%)	Total Phenolic Content (μg/g)	Antioxidant Activity (mg/100g)
Optimization (predicted values)	15	10	41.63	3189.2	40.37
Validation (determined values)	15	10	38.31	2842.33	37.66

MIC: minimum inhibitory concentration; CCEOC: *C. citratus* essential oil concentration.

## Data Availability

Data are available on request from the author(s). The datasets generated during the experiment that support the findings of this study are available from the corresponding authors upon reasonable request.
